# Life at new extremes: Integrating stress physiology and the bio‐exposome in the Anthropocene

**DOI:** 10.1111/nyas.15355

**Published:** 2025-05-14

**Authors:** David Costantini, Simone Messina, Manrico Sebastiano, Valeria Marasco

**Affiliations:** ^1^ Department of Ecological and Biological Sciences University of Tuscia, Largo dell'Università snc Viterbo Italy; ^2^ Behavioural Ecology & Ecophysiology Group, Department of Biology University of Antwerp Wilrijk Belgium; ^3^ Research Institute of Wildlife Ecology, Department of Interdisciplinary Life Sciences University of Veterinary Medicine Vienna Vienna Austria

**Keywords:** antioxidants, bio‐exposome, climate changes, disease, environmental pollutants, global changes, health, One Health, oxidative stress, physiological damage

## Abstract

Conventional physiological research has focused on elucidating the endogenous mechanisms that underly the adaptations of species to life in extreme habitats, such as polar regions or deserts. In this review article, we argue that even habitats that are not considered extremes are facing unpredictable, rapid, and strong modifications due to human activities that expose animals to novel extreme conditions. Thus, physiological research on these animals can offer insight on the role of physiological plasticity in driving their resilience and adaptation. To this end, we discuss how stress physiology (with a particular focus on oxidative stress) has a central role in mediating the interaction between the exposome (measure of all the environmental exposures of an individual in a lifetime) and cellular processes (bio‐exposome) in the contexts of relevant extreme anthropogenic changes to the habitat conditions. We also provide concrete examples on the relationship between oxidative stress and the bio‐exposome in free‐living animals, and how this research can be relevant to human health. Finally, we propose future research directions integrating the bio‐exposome and the One Health framework to achieve a holistic understanding of the proximate mechanisms underlying individual responses to extreme anthropogenic environmental changes.

## Graphical abstract

We propose to expand the concept of extreme environments to encompass those that are profoundly altered by climate and global changes, highlight the critical role of stress resilience in understanding how the exposome interacts with intracellular processes (bio‐exposome) in wildlife, emphasize how such interactions are relevant to the human bio‐exposome, and explore future research avenues that integrate the bio‐exposome with the One Health framework.

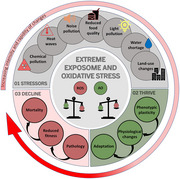

## LIFE AT THE LIMITS: PHYSIOLOGICAL ADAPTATIONS TO THRIVE IN THE ANTHROPOCENE

Living life at the limits of environmental abiotic conditions (e.g., those of polar regions or deserts) has required the evolution of sophisticated molecular and cellular adaptations that physiological research has classically investigated.^1^, ^2^ In the Anthropocene, habitats that are not classically considered extremes are being frequently exposed to severe human‐driven environmental stressors, such as chemical spills, degradation or transformation of landscapes, heatwaves, droughts and floods, and light and noise pollution. In many cases, these anthropogenic modifications of habitats are so harsh in terms of the rate at which they occur or their magnitude (e.g., heatwaves), that we feel it is reasonable (if not necessary) to expand the concept of extreme to include those environments that are significantly altered or influenced by human activities. This novel conceptual framework would offer models to (i) address how and why certain molecular and cellular adaptations emerge in response to the rise of extreme environmental conditions, (ii) provide a mechanistic understanding of the variation among conspecific individuals and species in their capacity to cope with such changes, (iii) identify the physiological endpoints that are diagnostic of organismal health and/or fitness outcomes, and (iv) inform predictions about population consequences.^3^ The conservation status of a given species is traditionally assessed by monitoring the population abundance over time, on a regional, national, or international scale. Developing mechanistic relationships between population declines and associated physiological processes is a major challenge for conservation physiologists.^4^ To this end, we recognize that it is desirable to gather relevant insight into the potential interactive effects of multiple extreme environmental stressors that organisms are exposed throughout their lifespan, from conception to death.

**FIGURE 1:**
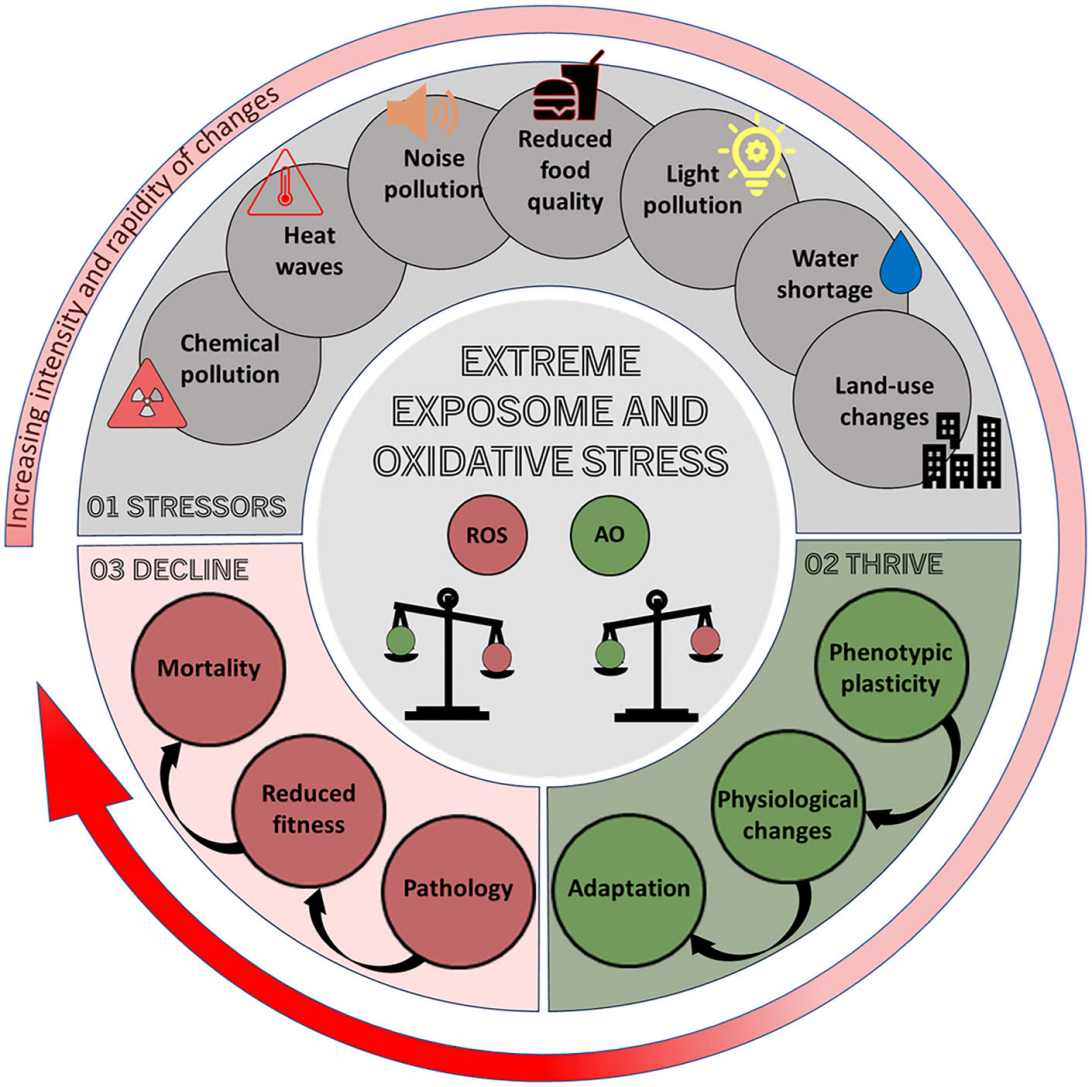
Illustration of different types of extreme anthropogenic environmental changes that shape the exposome and of their potential effects on individual health through the generation of oxidative stress. ROS = reactive oxygen species; AO = enzymatic and non‐enzymatic antioxidants.

The concept of exposome is particularly useful in dissecting the complexity of human‐modified environments and improving our approach to exposure assessment. The exposome includes the lifetime exposure of organisms to internal and external biological, chemical, and physical agents (with the exclusion of genetic heritable factors) that can impact on health.^5^, ^6^, ^7^ The exposome framework has originally been conceived to improve assessment of individual exposures through the development of biomarkers of organismal health. Recently, Minnis et al.^8^ proposed to move beyond the classic exposome, adopting the framework of bio‐exposome, which would also enable to elucidate endogenous mechanisms or causality (Figure 1). Implicit in the bio‐exposome is the importance of having an integrated approach to research, because a large number of important chronic diseases, accelerated ageing or impaired reproductive outcomes in humans likely result from the exposure to a combination of multiple stressors (acting through cumulative or synergistic effects) and their interaction with the genome.^9^ Emphasis is therefore placed in understanding how interactions between multiple environmental stressors contribute to fine tune, or alter, the bio‐exposome profile of an individual throughout the life course, but also to identify sensitive time windows or life stages when possible negative health outcomes might be effectively prevented or diminished.

In various scenarios, changes of the conditions that characterize an exposome occur at a rate that is too rapid for genetic adaptation to kick in, particularly in those species with long generation times. It is here that physiological plasticity, especially during pre‐ and postnatal development, is predicted to generate short‐term compensatory responses that enable environmentally induced adjustments of the phenotype, increasing the probability of the organisms to survive and reproduce.^10^, ^11^ A wealth of evidence shows that a variety of environmental stressors (e.g., associated with maternal, social, and nutritional conditions) during early development can generate a lasting molecular and neurophysiological memory, shaping the adult phenotype. This probably occurs through the pleiotropic effects of glucocorticoid hormones on cellular and metabolic homeostasis,^12^, ^13^ potentially influencing multiple generations.^14^, ^15^ The evolutionary role of these long‐lasting effects, particularly whether they foster coping mechanisms and resilience within a single generation or across multiple generations, remains an open debate.^16^

The plasticity of the physiological machinery might play a key role in driving the capacity of animal populations to face the new and often extreme environmental conditions of the Anthropocene. For example, decades of research have elucidated the intricate mechanisms linking endocrine pathways regulating the stress response (especially the hypothalamic‐pituitary‐adrenal (HPA) axis), immune function, or cellular energetics, with individual health or fitness outcomes.^17^, ^18^ In recent times, there has been increasing recognition in evolutionary ecology and ecophysiology that the need for organisms to regulate the cellular generation and pro‐oxidant activity of reactive oxygen species (ROS) has played a significant role in shaping their evolution. ROS is a collective term that includes the oxygen‐derived free radical and nonradical chemicals, such as superoxide anion, hydroxyl free radical, hydrogen peroxide (H_2_O_2_), and hypochlorous acid (HOCl).^19^ The action of ROS is counteracted by antioxidants, which include any mechanism, structure, and/or substance that prevents, delays, removes, or protects against oxidative nonenzymatic chemical modification (damage) to target molecules, including DNA and proteins.^20^ The levels of ROS and antioxidants in cells and tissues determine the oxidative status, while their interaction governs the rate of macromolecule oxidation. ROS‐induced oxidation is what we call oxidative damage, which is considered an important proxy to estimate the level of oxidative stress. There is substantial evidence across a wide range of taxa that oxidative stress plays a central role in biological and ecological processes.^21^ Changes in oxidative status in response to unpredictable or changing environmental conditions have been linked with changes in key traits, such as growth, reproduction, and longevity.^22^, ^23^, ^24^ Thus, we hypothesize that organismal oxidative stress might contribute to determine the chances of species or conspecific populations and individuals, to perish, cope, or even thrive in anthropogenic extreme environments.

In this review article, we focus exclusively on extreme anthropogenic changes to highlight the central role of stress physiology (with a particular focus on oxidative stress) in mediating the interaction between the external exposome and intercellular processes (as a single, integrated bio‐exposome as defined in Ref. 8). Although we focus on pathways that regulate the oxidative status of cells, our points are also relevant for other physiological mechanisms underlying individual differences in the functioning of the stress responses, thus our considerations are of broad application. We discuss the importance of oxidative status regulation in the context of four key anthropogenic extreme changes of the habitat conditions: pollutant exposure, dietary shifts due to anthropogenic food waste consumption, land‐cover alteration, and climate change. Then, we present a critical appraisal of what this research on nonconventional animal models may teach us about bio‐exposome and human health. Finally, we discuss avenues of research to integrate the bio‐exposome within the One Health framework.

## THE KEY ROLE OF OXIDATIVE STRESS IN UNDERSTANDING ENVIRONMENTAL POLLUTION

Elevated levels of nonessential trace elements, the massive use of pesticides, and the extensive release of industrial and nonindustrial chemicals into the environment, including plastics, can disrupt organismal homeostasis, leading to oxidative stress. Some chemicals directly impact on the oxidative status by affecting the rate at which ROS are generated or disposed of. For example, Eurasian tree sparrows *Passer montanus* in polluted areas exhibit reduced antioxidant levels,^25^ Arctic black‐legged kittiwakes *Rissa tridactyla* exposed to higher concentrations of perfluoroalkyl and polyfluoroalkyl substances (PFAS) show higher levels of oxidative damage,^26^ and black‐vented shearwaters *Puffinus opisthomelas* with higher mercury concentrations in blood have lower antioxidant levels.^27^ Further work found decreased activity of antioxidant enzymes and down‐regulated expression of antioxidant‐related genes in fish that were experimentally exposed to pesticides.^28^ Pollutants can also indirectly cause oxidative stress by eliciting pro‐inflammatory intracellular signaling cascades leading to the activation of immune cells,^29^ which are relevant generators of ROS.^30^

On the other hand, other studies found evidence for rapid upregulation of antioxidants to withstand the pro‐oxidant action of pollutants. For example, intertidal copepods *Tigriopus japonicus* exposed to silver, arsenic, and copper, show a remarkable capability in coping with the pro‐oxidant action of these heavy metals by upregulating the activity of several antioxidant enzymes.^31^ Similarly, zebrafish embryos exposed to low doses of fungicides enhanced the activity of the antioxidant enzymes superoxide dismutase and glutathione‐*S*‐transferase.^32^ Finally, striped rockcods *Trematomus hansoni* experimentally exposed to inorganic pollutants increased the expression levels of metallothioneins,^33^ a group of proteins that, by binding to metal ions, protect cells against oxidative stress.^34^

**FIGURE 2:**
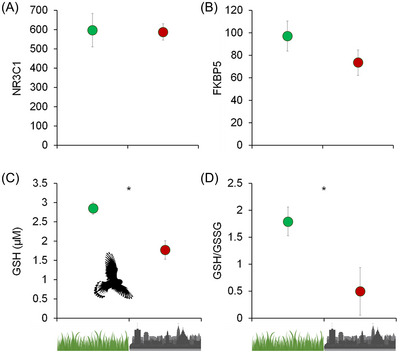
Regulation of oxidative status plays a key role in the maintenance of homeostasis across contrasting habitats. Natural and urban nestlings of the common kestrel *Falco tinnunculus* show similar levels of expression of the genes (A) NR3C1 (encodes the glucocorticoid receptor) and (B) FKBP5 (regulates the expression of NR3C1) in blood, suggesting similar stimulation of the hypothalamic–pituitary–adrenal axis (from Isaksson et al.^146^); by contrast, urban kestrels show (C) lower blood concentration of the antioxidant glutathione (GSH) and (D) smaller ratios of reduced glutathione onto oxidized glutathione (GSH/GSSG) than natural kestrels, suggesting higher oxidative stress in urban kestrels (from Liguori et al.^139^). Values are shown as mean and standard deviation. Gene expression data are expressed as read counts.

Current evidence suggests that the sensitivity to pollution is also influenced by a combination of factors that reflect the bio‐exposome, such as habitat, the species‐specific mechanisms of defense, and the age and/or life‐history stage during which an organism is exposed to pollutants.^35^, ^36^, ^37^ For example, previous meta‐analytic work, encompassing several species of vertebrates and invertebrates showed that the pro‐oxidant effects of pollutants are stronger in adults than in juveniles, probably because many pollutants accumulate with age and can cause pathological effects later in life.^36^ Moreover, the meta‐analysis showed that a change of glutathione concentration (increase or decrease) was the strongest consequence of exposure to pollutants in animals^36^ (Figure 2). This is particularly relevant given the widespread occurrence of glutathione in organisms, including plants and animals, and its importance for cell homeostasis.^19^ Oxidative stress can therefore represent a critical indicator of the capability of an organism to cope with environmental pollutants and thus, the efficiency of the antioxidant barrier to influence the adaptation of animals to pollution‐induced oxidative stress (e.g., mercury chloride^38^, ^39^).

Indeed, when environmental contaminants generate excessive ROS that overwhelm the antioxidant defenses, the resulting oxidative imbalance can ultimately impair physiological functions critical for fitness‐related functions, such as reproduction. For example, mice exposed to PFAS showed oocyte apoptosis, most likely related to a spike in ROS levels in the ovary.^40^ Similarly, PFAS exposure in mice compromised the viability of spermatogonial cells through increased ROS and malondialdehyde (a marker of oxidative damage) generation.^41^ Thus, the link between environmental pollution and oxidative stress suggests that the regulation of oxidative status plays a central role in influencing the evolution of adaptive coping mechanisms of natural animal populations to chemical contamination of their habitats,^42^ so that assessment of oxidative status markers is indispensable to elucidate the ways through which the exposome influences organismal health outcomes.

## THE LURE OF LANDFILLS: HOW GARBAGE IS MODIFYING THE PHYSIOLOGY AND LIFE HISTORIES OF ANIMALS

It is estimated that as much as one‐third of all food produced is wasted, likely ending up in landfills.^43^ As it stands, global municipal waste is predicted to increase to 2.4 billion tons by 2050, more than double the current predictions for population growth. Thus, this phenomenon is set to keep growing. Not only anthropogenic organic wastes attract a myriad of small organisms, but also many larger vertebrates, especially birds and mammals. Nowadays, various species of gulls, corvids, squirrels, and bears, are known to be opportunistic or specialized exploiters of landfills as a new food resource.^43^

A key question that has just started being investigated is what feeding on anthropogenic organic wastes means for the physiology of wildlife, and what the implications are for their life histories and, in broader terms, how it determines health outcomes. Free‐living animals feeding on human waste have the advantage of predictable and abundant food resources that may help them to cope with seasonal harshness as shown in urban gray squirrels^44^ (see also the meta‐analysis by Chamberlain et al.^45^). Studies on birds indicate that feeding on anthropogenic food waste often translates into increased population breeding outcomes,^46^ improved nutritional status,^47^ and reduced energy expenditure during the demanding phases of reproduction.^48^ In the migratory white stork *Ciconia ciconia*, it has been suggested that feeding on anthropogenic food waste, a phenomenon largely documented across most breeding populations in Western Europe (the process is at the beginnings in Eastern Europe^49^), played a key role for the recovery of declining populations.^50^, ^51^

High population densities might not necessarily translate into healthy populations if the quality of anthropogenic food is low. Animals foraging on human waste often consume a diet drastically different from their natural food sources, for example, fattier, richer of processed nutrients, likely deficient in vitamins and antioxidants due to their fast rates of degradation, and enriched with preservatives and other industrial chemicals.^52^ Various studies across different species have consistently documented an increase in plasma cholesterol, glucose, and triglycerides in individuals living in more urbanized environments or in association with anthropogenic organic wastes^47^, ^53^, ^54^, ^55^ (but see Ref. 56). Although some of the changes in blood chemistry documented in “landfill‐friendly wildlife” might confer some health advantages over the short‐term (e.g., due to good nutritional status and energy reserves^47^, ^57^), they might become detrimental over longer time scales, depending on the chronological age at exposure and the overall timing of exposure throughout an individual's lifetime. For example, research in humans and laboratory rodents indicates that the impact of sustained energy‐rich unhealthy diets, especially during the early pre‐ and postnatal stages of growth, can be particularly profound and long‐lasting, leading to higher metabolic risks, anxiety, depressive‐like behaviors, and impaired cognition.^58^, ^59^ There can be obvious additional disadvantages arising from wildlife feeding on human wastes, including ingestion of hazardous items and pollutants, transmission of infectious diseases and new emerging diseases, and, possibly, acquisition of resistance to antibiotics.^60^, ^61^

Oxidative stress is likely to be a critical endogenous mechanism linking the impact of anthropogenic food waste to the bio‐exposome of organisms given that (i) excessive caloric intake leads to elevated inflammation and metabolic activity, thus increasing the generation of ROS;^62^ (ii) exposure to many contaminants associated with waste decomposition (including heavy metals, radioactive pollutants, endocrine disrupting compounds, and polycyclic aromatic hydrocarbons, ubiquitously present in landfill leachate) have pro‐oxidant and pro‐inflammatory effects as a common denominator, which can lead to increased oxidative damage and altered modulation of the antioxidant system;^37^, ^63^ and (iii) the ingestion of degraded food can lead to lower circulatory levels of dietary‐acquired compounds with antioxidant capacities (e.g., vitamin E and carotenoids).^52^ Additionally, garbage accumulation can act as an interface between humans, animals, and the environment leading to increased intraspecies and interspecies rates of contact, accumulation of pathogens, and higher risks of zoonotic and infectious diseases, which is of concern not only for wildlife health, but also for public health in general.^61^

**FIGURE 3:**
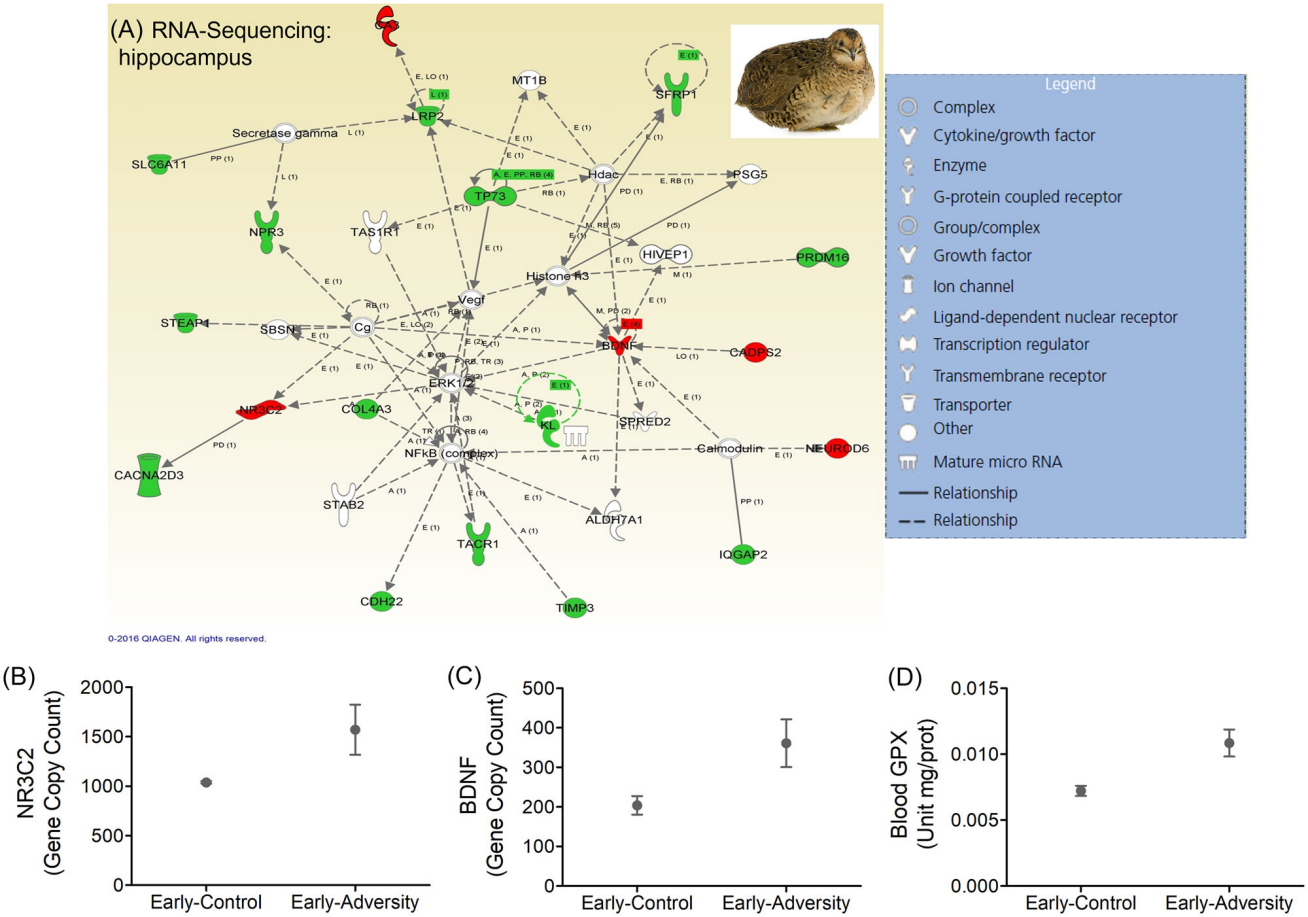
Adversity early in life in the Japanese quail (mimicked through physiological elevation of corticosterone hormone during prenatal development, through egg injection, and early postnatal growth, through oral administration) led to subsequent transcriptome changes in the brain and markers of oxidative stress into adulthood. In (A) is functional gene expression network (generated by Ingenuity Pathway Analysis software, IPA) with downregulated genes (green) and upregulated genes (red) in response to early‐life adversity in hippocampal‐derived tissues of adult quail; the network is displayed with nodes (i.e., genes) and edges (i.e., biological interactions among nodes); in white, the not user‐specific genes added into the network as a result of interactions with the submitted (i.e., user‐specific) genes. Solid lines connecting the genes indicate direct interactions between the nodes and dashed lines imply indirect interactions. Expression levels of (B) NR3C2 (which encodes the mineralocorticoid receptor regulating HPA axis functioning) and (C) BDNF (encoding brain‐derived neurotrophic factor, known to promote spatial memory and cognition abilities) were higher in adult quail exposed to early‐life adversity (graphs B and C data from Marasco et al.^12^). Adult quails treated in early life with corticosterone also showed upregulation of (D) blood glutathione peroxidase (GPx) compared to controls (data from Marasco et al.^135^). Values are shown as mean and standard error.

Relatively limited studies so far have directly investigated this topic, and most of the research has been carried out in free‐living birds. Surprisingly, storks fed with food from landfills during their postnatal growth stages (as nestlings) did not show higher levels of oxidative damage compared to nestling storks fed with a more natural diet, possibly due to enhanced levels of endogenous antioxidants.^47^ This effect may have emerged because of improved resilience through hormesis (low‐dose stimulation of organismal functions), or through programming (inoculation‐like effects) during early life when physiological plasticity to adverse environmental exposures is predicted to be highest^42^, ^64^ (see also Figure 3). However, the capacity to buffer dietary‐related stressors may be offset by additional exposomic challenges, such as climate changes.^65^

Research so far investigated effects over short‐term scales, and mostly at the population rather than individual level (but see Ref. 66). Longitudinal long‐term studies, however, are likely to be much more enlightening as the bio‐exposome of an individual encompasses gene‐environment interactions over the lifetime, and the consequent health effects of such interactions may be modulated by concurrent ageing and other factors of physiological deterioration. For example, in free‐ranging black bears *Ursus americanus*, consumption of human foods reduced hibernation periods and induced accelerated rates of telomere loss.^67^ As telomere loss reflects within‐individual differences in survival and ageing rates across many bird and mammalian species^68^, ^69^, ^70^, ^71^ and is a marker strictly associated with oxidative stress,^72^ these results call for more long‐term studies to fully understand the bi‐directional influences among dietary shifts, the processes regulating the molecular machinery, and the resulting morbidity outcomes. It could be particularly relevant to address these questions in seasonal organisms, which need to rapidly store energy in preparation to migration or hibernation, and thus could be particularly susceptible to the potential long‐term fitness costs of human food waste consumption.

## CHANGING LANDSCAPES: THE GOOD, THE BAD, AND THE UGLY

Environmental pollution and dietary changes are often linked to anthropogenically altered habitats. Secondary forests, forest fragments, and different forms of land conversion, such as agricultural fields, pastures, and urban areas, are main drivers of biodiversity loss^73^ and powerful forces of selection imposing physiological adjustments to organisms facing the new environmental conditions.^74^ One relevant result of such extreme forces of selection is typically a decline of species diversity in favor of fewer, more adaptable species, but the strength of such changes is dependent on the form and intensity of the land‐use change.^75^, ^76^, ^77^

The adaptation of species to changing habitats may be determined by ecological, morpho‐functional, and physiological traits. In particular, mechanisms of stress physiology, such as glucocorticoid hormones and oxidative status regulation, seem to play important roles as mediators of species responses to novel environmental conditions, because they underlie important fitness traits, including survival and reproduction.^78^, ^79^ In degraded habitats, energy mobilization induced by higher baseline levels of glucocorticoids may be necessary to support longer foraging trips.^80^, ^81^, ^82^ In turn, reduced food availability may affect the oxidative status of organisms through a reduced intake of dietary antioxidants and increased metabolic demands.^83^ Studies on the impact of tropical forest selective logging on understory birds found that higher levels of feather corticosterone and of blood antioxidant markers (i.e., nonenzymatic antioxidant capacity, glutathione, and glutathione peroxidase) were associated with lower population abundances.^84^, ^85^ These results suggest that the regulation of the HPA‐axis might concur with a higher investment in certain antioxidant mechanisms (e.g., glutathione cycle) resulting in a trade‐off between self‐maintenance and reproductive or developmental metrics, which translates into population effects. The results of experimental studies on birds indicate that higher baseline glucocorticoid levels in early life reduce the efficiency of mitochondria in producing adenosine triphosphate (ATP).^86^ This reduced production of energy might limit the synthesis of endogenous antioxidants, which requires ATP.^19^

Further important evidence on the role of oxidative status regulation in the process of adaptation to novel extreme environmental conditions comes from studies on conspecifics from rural and urban areas (see also Paragraph 6). Cities are environments where organisms are exposed to an array of novel, potentially extreme, conditions, such as air pollution, noise and light pollution, heatwaves, and easy access to food of low nutritional quality. The impact of urban life on the organismal physiology may be strong, and HPA‐axis responsiveness and protection against oxidative stress are important mechanisms driving acclimation or adaptation to urban habitats.^87^, ^88^, ^89^, ^90^, ^91^, ^92^ For example, increasing urban cover was associated with decreased nonenzymatic antioxidant capacity and immune response to a mimicked bacterial infection in nestlings of a South African raptor (i.e., black sparrowhawk, *Accipiter melanoleucus*), suggesting that urbanization impairs aspects of immune function by limiting the capacity of nestlings to prevent oxidative stress.^93^

Finally, land‐use change may influence exposure to parasites and the local abundance of their vectors, exposing animals and humans to a higher risk of infection and new zoonosis.^94^, ^95^, ^96^ Hemosporidian infections, which include the vector‐transmitted genus *Plasmodium* among others, can have either lethal or sublethal consequences by inducing increased metabolic demands and physiological costs to control the infection.^97^, ^98^ In birds, chronic parasitism reduces survival and reproductive success.^99^, ^100^ Lower levels of parasite burden are associated with diets rich in antioxidants.^101^, ^102^ Thus, habitat changes that reduce nutritional resources might negatively affect susceptibility to parasites, increasing the risk of infection.^103^ Furthermore, studies on humans and avian *Plasmodium* infections indicate the redox cycle of glutathione as one major antioxidant mechanism involved in the physiological response to infection.^104^, ^105^, ^106^

## PHYSIOLOGICAL PLASTICITY TO COPE WITH EXTREME GLOBAL CLIMATE CHANGES

Climate change is an increasingly direct, or indirect, driver of the biodiversity crisis.^107^ Model predictions on future climate scenarios have suggested that many species will substantially reduce their distribution, or even become extinct globally.^108^, ^109^ Other species may instead gain benefits from climate change, and move into new habitats expanding their range.^110^ Species at higher risk of extinction due to climate change are those forced to move because physiologically limited for acclimation, but whose dispersion is prevented by geographic barriers of natural or anthropic origin (i.e., land‐use changes^111^). Moreover, many species facing land‐use changes may be already close to their climatic tolerance limits, thus they might be more vulnerable to extreme climatic events (e.g., heatwaves, drought).^112^, ^113^, ^114^ On the other hand, those species that have high capacity for physiological plasticity and thermal acclimation might thrive in the Anthropocene.

Ectothermic and homeothermic animals substantially differ in their thermoregulatory mechanisms. Ectotherms are strongly dependent on the ambient temperature due to their incapability to use metabolic heat to thermoregulate. Rising temperatures can push ectotherms outside their physiological optima, closer to their thermal tolerance limits or even above, in the stressful range.^115^ By influencing the rate of metabolism and biochemical reactions, environmental temperature affects organismal fitness and the abundance of ectothermic species.^116^ Differently, homeotherms can maintain a stable body temperature (*T*_b_) within a range of ambient temperatures (*T*_a_), called thermoneutral zone, within which the resting metabolic rate is independent of *T*_a_. Homeothermic species are better buffered against rising temperatures than ectotherms by virtue of their high normothermic *T*_b_. However, when *T*_a_ rises above the thermoneutral zone, the organism needs to spend energy to thermoregulate, or it enters a hyperthermic state with potential lethal or sublethal effects.^117^ Despite such important physiological differences, rising temperatures and heatwaves can negatively impact the fitness of both ectotherms and endotherms, in some cases causing mass mortality episodes.^118^, ^119^

Thermal stress induces cellular oxidative stress in ectothermic and homeothermic animals.^120^, ^121^, ^122^ Oxidative stress may be a key mediator of the fitness of organisms exposed to thermal stress, given that the resultant tissue degradation might influence reproductive performance, growth patterns, senescence rate, and survival.^123^, ^124^, ^125^ For example, by increasing oxidative lesions to the guanine of telomeric DNA, ectotherms living at temperatures close to their thermal limits, or exposed to intense heatwaves, undergo accelerated senescence rates.^126^, ^127^ While a causal relationship between thermal environment, oxidative damage, and telomere shortening has been shown for ectotherms, we lack data on homeotherm species (but see Ref. 128). In response to increased oxidative damage, organisms induce the expression of endogenous antioxidant enzymes, which play a major role in cellular detoxification from ROS. The upregulation of antioxidant defenses, however, is energetically costly, and there is interindividual variation in the capability to face oxidative challenges induced by thermal stress. Loughland and Seebacher^129^ found that experimentally increased antioxidant capacities of mosquitofish *Gambusia holbrooki* reduced oxidative damage in individuals with low capacity for acclimation to cold temperatures, suggesting a causal relationship between thermal acclimation and oxidative stress.

Physiological plasticity has also the potential to buffer organisms from warming by reducing the thermal sensitivity of life‐sustaining processes and increasing physiological tolerances.^114^, ^130^ One mechanism through which physiological plasticity may act is through hormetic priming, which would enable organisms to maintain adequate levels of fitness under variable environmental conditions.^131^, ^132^ The hormetic model proposes that exposure to certain levels of environmental stress, below a critic threshold, would trigger stimulatory effects on the organism, resulting in individuals better able to cope with higher levels of that same stressor across life. Hormesis operates via several physiological and molecular mechanisms, but a pervasive role in hormesis is played by antioxidant mechanisms that reduce oxidative damage and prevent oxidative stress.^133^ Studies on homeothermic animals, such as birds, showed that early conditioning to heat stress primed individuals to cope with high ambient temperatures experienced later in life, increasing their resistance to oxidative stress.^134^, ^135^ Such effects could be mediated by elevated glucocorticoid hormone exposure during early life, as the actions of these hormones in organizing centrally regulated mechanisms of energy availability can be particularly profound and long‐lasting, and might be important promoters of subsequent physiological resilience^16^, ^86^, ^136^ (see also Figure 3). Though, whether hormesis or early life programming foster organismal resistance to extreme climatic events is a key unanswered question that requires future integrative research.

## STEPPING INTO THE WILD: WHAT ANIMALS TEACH US ABOUT BIOEXPOSOME AND HUMAN HEALTH

There is a significant link between the exposome and the increasing number of human health issues, such as reduced fertility, altered brain development, diabetes, allergies, or various types of cancer.^137^, ^138^ It is challenging to develop compelling evidence that alterations of certain physiological pathways due to environmental changes cause specific adverse health effects. Epidemiological evidence suggests that oxidative stress is a unifying component of many diseases, including neurodegenerative diseases, cancer, atherosclerosis, and chronic obstructive pulmonary disease, owing to its genotoxic effects and alteration of cellular redox signalling.^139^, ^140^ For example, Li et al.^141^ showed that oxidative stress plays a relevant role in ambient particulate matter–induced lung diseases. Similarly, Fiorito et al.^142^ found that chronic exposure to air pollution can lead to oxidative stress, which in turn activates a cascade of inflammatory responses, leading to increased risk of cardio‐ and cerebrovascular diseases. It has also been suggested that oxidative stress plays a role in the increased risk of adverse cardiovascular diseases in individuals exposed to organic pollutants, such as polycyclic aromatic hydrocarbons.^143^

Research on animals that live in urban and suburban environments provides a broad group of species that can prove excellent non‐conventional models to study the urban exposome and its potential consequences for humans. One reason for this lies in the evolutionary conservation of physiological mechanisms that regulate energy metabolism, including the cellular oxidative status and the HPA axis functioning in vertebrates. Second, those animal species that cohabit with us in urban areas are particularly valuable because they are being exposed to similar types of environmental stressors. Birds and mammals probably represent the vertebrates that most successfully adapted to synanthropic life in cites,^144^, ^145^ which makes them valuable sentinels of changing environments. For example, studies on birds show that the plasma concentration of glutathione in nestlings growing in urban environments was different from that measured in conspecific nestlings growing in nonurban environments, possibly indicating a different generation of ROS or synthesis of glutathione in animals living in urban areas^146^, ^147^ (Figure 2). A comparative study on birds found that rural populations had higher concentrations of two dietary antioxidants (vitamin E and carotenoids) in the liver than urban conspecifics, likely caused by differences in their diet.^148^ However, early experimental studies carried out under common garden conditions found that urban blackbirds *Turdus merula* had a reduced stress responsiveness^149^ and lower levels of blood oxidative damage^87^ compared to rural blackbirds. Moreover, a cross‐fostering experiment on great tits *Parus major* demonstrated that being raised in the urban environment triggers an increase of the antioxidant enzyme superoxide dismutase, regardless of the natal habitat.^150^ Studies on free‐living urban mammals are surprisingly underrepresented relatively to birds,^151^ but are hopefully on the rise.^152^ Thus, variation among species in resistance against or tolerance of oxidative stress might underlie their capability for adaptation to urban life, which is particularly relevant for humans owing to the potential pathological consequences of augmented oxidative stress.^138^, ^139^

As shown in Figures 2 and 3, and illustrated in the next paragraph, combining multiple markers of oxidative status with omics techniques that target the expression of and selection on genes that regulate stress resilience and oxidative status would provide a valuable approach to associate individual exposomes with health effects. This approach would also help to estimate the contribution of the various components that shape the exposomes, from environmental chemicals to abiotic and social factors.

## PROSPECTIVES AND FUTURE DIRECTIONS

### Functional exposomics: A tool to understand the totality of exposure–phenotype interactions

Omics characterization through the profiling of the genome, transcriptome, proteome, and metabolome at the individual scale is an extremely exciting opportunity to obtain more realistic signatures of individual bio‐exposomes in response to anthropogenic‐related new extremes. The successful proof of concept for this comes from recent research in wildlife showing that blood transcriptomics can reveal intraspecific molecular differences in relation to urbanization,^153^, ^154^ water pollution,^155^ elevational gradients,^156^ and disease dynamics.^157^ Omics datasets are of difficult interpretation, especially in species lacking informative annotation of their genomes and proteomes. To move the field forward, we encourage studies integrating omics assessment of exposed individuals with traditional measurements of physiological resilience (including but not limited to HPA axis maximal responsiveness and recovery to baseline, markers of oxidative damage and antioxidants, telomere length and dynamics), as well as direct observations of fitness and health outcomes. This approach would enable to link functional changes with mechanisms, moving from exposure association to causation. Through this approach we could potentially (i) estimate how different components of the exposome impact alone or in interaction with each other on the organism and (ii) identify mechanisms that are physiologically modifiable (e.g., cellular repair processes during specific age‐ or seasonal‐phenotypic states, such as hibernation^158^), or long‐lasting (e.g., through developmental programming effects of molecular stress physiology pathways in the brain;^12^ Figure 3) and specifically attributable to the emergence of adaptation or vulnerability.^159^

This approach would pave the way to innovative research horizons with a great significance for both basic and clinical research sectors at the interface of the life sciences and veterinary medicine. It is generally assumed that the magnitude of long‐lasting irreversible phenotypic effects of environmental stressors diminish with increasing age.^160^ However, responses to stress exposure can also emerge into adult life and generate long‐lasting fitness consequences.^23^, ^71^, ^161^ Thus, long‐term longitudinal study designs and minimally invasive tissue sampling (e.g., blood, fecal material, urine) are likely to be the new methodological frontiers to identify effective strategies to prevent major causes of morbidity and reduced fitness and/or health outcomes across the lifespan of an individual.

### One Health meets the bio‐exposome

The bio‐exposome framework shows remarkable synergies with the One Health framework, the latter being defined as a collaborative and transdisciplinary approach that recognizes the interconnections among humans, wildlife, and their shared environment with the goal of achieving optimal health outcomes. Organisms of each health domain (including environment, animals, and humans) are interconnected to each other, indicating that any effect of the bio‐exposome on a particular group of conspecific individuals can scale up to the species, community or even ecosystem level (see Ref. 162 for an appraisal on methodologies). This integrated and multidimensional approach is increasingly achievable thanks to the rapid advances in the omics technologies and big data analytics, such as machine learning algorithms and animal tracking approaches (recently reviewed in Ref. 163). Yet, solely technology advances will not suffice if scientists are not encouraged to collaborate across boundaries. System science and system thinking can provide a crucial “doorway” in this context.^164^

## CONCLUSIONS

In this review article, we have argued that we need more basic physiological research on animals that live in habitats that are classically not considered extremes, but that are currently being exposed to unpredictable, rapid, and strong anthropogenic stressors that generate novel extreme conditions. In this framework, we point out the relevance of the integration between bio‐exposome and oxidative status to address the organismal consequences and the potential interactions of impacted organisms. The maintenance of the organismal oxidative status homeostasis represents a widespread and highly conserved need across animals, including humans. This is because any perturbations of the oxidative status can potentially result in detrimental consequences for the organism, such as increased risk of developing disease, reduced fertility, or faster rate of cellular senescence. This link between oxidative status and health or fitness is particularly pertinent under the current rate and scale of extreme human‐induced changes of habitats. The mechanisms through which organisms maintain an optimal oxidative status against environmental perturbations might hold one of the keys to unlock novel insights into the impact of extreme anthropogenic events on wildlife and human health, and the animal–human–environment interfaces. By considering the link between the bio‐exposome and stress physiology, we would have a better understanding of the proximate mechanisms that underlie the impact of different types of exposomes on the organism, and of the reasons why some species flourish or perish in the new extremes of the Anthropocene.

The ability to maintain a balanced cellular environment and to mitigate oxidative stress by either minimizing the impact of oxidant stimuli or by enhancing antioxidant defenses represents important components of these adaptive coping mechanisms. Thus, they might be key mechanisms underlying the chances of animals to thrive in a world where extreme anthropogenic changes are predicted to increase. Future research should therefore focus on uncovering the genetic and epigenetic foundations of these adaptations, exploring the bio‐exposome across a diverse range of species to identify common stress response mechanisms. Additionally, a comprehensive approach combining omics techniques, multimarker approaches, and species‐wide association studies would be needed to evaluate the additive, synergistic, and antagonistic effects of the various components that shape the bio‐exposome. We have many exciting challenges ahead, and we hope this article will contribute to encourage holistic approaches to identify the key proximate mechanisms linking life at the new extremes to population and environmental health.

## AUTHOR CONTRIBUTIONS

D.C., S.M., M.S., and V.M. conceived the idea and wrote the manuscript.

## CONFLICT OF INTEREST STATEMENT

The authors declare no conflicts of interest.

## PEER REVIEW

The peer review history for this article is available at https://publons.com/publon/10.1111/nyas.15355.

## Data Availability

Data sharing is not applicable to this article as no datasets were generated or analyzed during the current study.
